# Effects of three silane primers and five adhesive agents on the bond strength of composite material for a computer-aided design and manufacturing system

**DOI:** 10.1590/1678-7757-2017-0342

**Published:** 2018-04-18

**Authors:** Ayano Shinohara, Yohsuke Taira, Michino Sakihara, Takashi Sawase

**Affiliations:** 1 Nagasaki University, Graduate School of Biomedical Sciences, Department of Applied Prosthodontics, Japan.

**Keywords:** Bond strength, CAD-CAM, Composite resin, Adhesive

## Abstract

**Objective:**

The objective of this study was to evaluate the effects of combinations of silane primers and adhesive agents on the bond strength of a composite block for a computer-aided design and manufacturing system.

**Material and Methods:**

Three silane primers [Clearfil Ceramic Primer (CP), Super-Bond PZ Primer (PZ), and GC Ceramic Primer II (GP)] were used in conjunction with five adhesive agents [G-Premio Bond (P-Bond), Repair Adhe Adhesive (R-Adhesive), Super-Bond D-Liner Dual (SB-Dual), Super-Bond C&B (SB-Self), and SB-Dual without tributylborane derivative (SB-Light)]. The surface of a composite block (Gradia Block) was ground with silicon carbide paper. After treatment with a silane primer, a adhesive agent was applied to each testing specimen. The specimens were then bonded with a light-curing resin composite. After 24 h, the shear bond strength values were determined and compared using a *post hoc* test (α=0.05, n=8/group). We also prepared control specimens without primer (No primer) and/or without adhesive agent (No adhesive).

**Results:**

PZ/SB-Dual and GP/SB-Dual presented the highest bond strength, followed by GP/P-Bond, CP/SB-Dual, CP/R-Adhesive, No primer/SB-Dual, GP/R-Adhesive, CP/P-Bond, No primer/R-Adhesive, PZ/R-Adhesive, CP/SB-Self, PZ/P-Bond, PZ/SB-Self, and GP/SB-Self in descending order of bond strength. No primer/P-Bond, No primer/SB-Self, and all specimens in the SB-Light and No adhesive groups presented the lowest bond strengths.

**Conclusion:**

A dual-curing adhesive agent (SB-Dual) containing a tributylborane derivative in combination with a silane primer (GP or PZ) presents a greater bond strength between the composite block and the repairing resin composite than the comparators used in the study.

## Introduction

The advent of computer-aided design and computer-aided manufacturing (CAD/CAM) systems, the use of highly filled resin composite materials became increasingly common for crown restorations^16^. Despite the improvement of the mechanical properties of composite blocks for CAD/CAM systems over time^8^ ^,^ ^13^, partial fractures still occur occasionally on restorations in the oral environment. Strong bonding between the machine-milled composite blocks and the resin composite veneering materials is required to repair defects in resin composite restorations or to modify the esthetics of the monochromatic composite blocks using the layering technique^15^.

Ceramic repair systems and pre-treatment agents have improved the bond strength of resin-based materials to composite blocks^4^ ^,^ ^6^. Certain studies recommend to treat the surface with silanes^1^ ^,^ ^5^ ^,^ ^19^ ^,^ ^20^, while others claim that silanization techniques do not increase bond strength^22^ ^,^ ^23^. When a silane primer and an adhesive agent were applied to the surface of a composite block, the bond strength was greater than that obtained through the use of a lithium disilicate glass ceramic or a feldspar ceramic^3^. Studies show that the combined use of a silane primer and a lightcuring adhesive agent is effective to increase the bond strength between the layers of a resin composite^11^ ^,^ ^12^. However, there is little information available on the effect of the type of polymerization initiator contained in the adhesive agent on the bond strength.

Masuhara^14^ (1969) developed a self-curing adhesive agent initiated with tributylborane (TBB) for dental treatment. The features of polymerization initiated by TBB are fundamentally different from those of the photoinitiators upon postpolymerization and interfacial initiation of polymerization^10^ ^,^ ^21^. Super-Bond D-Liner Dual (Sun Medical Co., Moriyama, Japan) is a commercially available adhesive agent that uses a dual-curing system containing a photoinitiator and TBB. Despite the reports of TBB being a useful initiator component^9^ ^,^ ^17^, it is not clear if dual-curing systems provide an added advantage vis-à-vis the bond strength of a composite block.

The objective of this study was to evaluate the bond strength between a composite block and the light-curing resin composite comprising five different adhesive agents (three light-curing systems, one self-curing system, and one dual-curing system) in conjunction with three silane primers. The null hypothesis was that there is no significant difference among the bond strengths for the different combinations of the adhesive agents and silane primers.

## Materials and methods

### Shear bond strength tests

**Figure 1:**
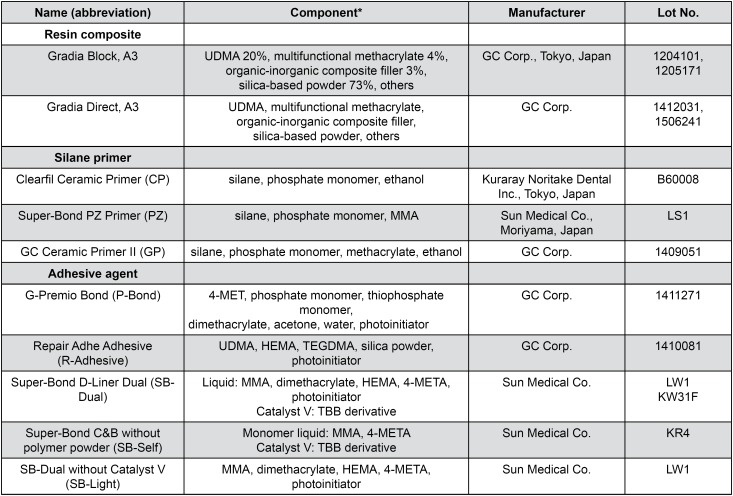
Resin composites, silane primers, and adhesives agents used for shear bond strength tests *UDMA: urethane dimethacrylate, MMA: methyl methacrylate, 4-MET: 4-methacryloyloxyethyl trimellitic acid, HEMA: 2-hydroxyethyl methacrylate, TEGDMA: triethyleneglycol dimethacrylate, 4-META: 4-methacryloyloxyethyl trimellitate anhydride, TBB: tributylborane

**Figure 2:**
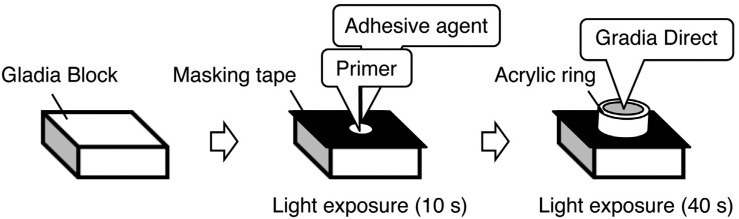
Schematic illustration of the bonding procedure

The three silane primers (CP, PZ, and GP) and five adhesive agents (P-Bond, R-Adhesive, SB-Dual, SB-Self, and SB-Light) used are listed in Figure 1. The testing specimens consisted of 192 rectangular specimens (8×10×3 mm) that were cut from a composite block (Gradia Block, A3, GC Corp., Tokyo, Japan) using a diamond saw (IsoMet Low Speed Saw, Buehler, Lake Bluff, IL, USA), they were divided into 24 groups (15 combinations of 3 primers and 5 adhesive agents and 9 controls) of 8 specimens each. All specimens were ground with 600-grit silicon-carbide abrasive paper (BuehlerMet2, Buehler, Lake Bluff, IL, USA), rinsed by spraying with water for 10 s, and air-dried. We attached a piece of masking tape with a circular hole of 2 mm in diameter to the surface of each specimen to delineate the bonding area (Figure 2). One microliter of each of the silane primer and adhesive agent was then applied to the specimens with a micropipette (Eppendorf AG, Hamburg, Germany) and gently air-blown. Except for SB-Self, the adhesive agents used were light-cured for 10 s using a light- emitting diode handpiece (power density 1,000 mW/ cm^2^; Pencure, J. Morita MFG. Corp., Tokyo, Japan). We also prepared control specimens without primer (No primer) and/or without adhesive agent (No adhesive).

We placed an acrylic ring (internal diameter: 4 mm, height: 2 mm) on the specimen and filled it with a light-curing resin composite (Gradia Direct, A3, GC Corp., Tokyo, Japan). The resin composite was then light-cured for 40 s. The specimens were stored in the atmosphere for 30 min and then immersed in water at 37°C for 24 h. Each specimen was embedded in an acrylic resin mold and placed in a shear-testing device (No. ISO/TR11405, Wago Industrial Ltd., Nagasaki, Japan). The shear bond strength of each specimen was determined using a universal testing machine (AGS-10kNG, Shimadzu Corp., Kyoto, Japan) at a cross-head speed of 0.5 mm/min.

The debonded surfaces of all specimens were observed with an optical microscope (SMZ-10, Nikon Corp., Tokyo, Japan) at a magnification of x20 to assess bond failure. Failure modes were categorized as adhesive failure at the interface between the composite block and veneered resin composite (Ad) and a combination of Ad and crack propagation (Cr) inside the composite block (Ad/Cr).

### Scanning electron microscopy (SEM)

In addition, a composite block was ground with 600-grit silicon-carbide abrasive paper, its surface was sputter-coated with gold (Ion Coater IB-3, Eiko Engineering Co. Ltd., Hitachinaka, Japan), then we observed it using a scanning electron microscope (JCM- 6000Plus, JEOL Ltd., Tokyo, Japan) at magnifications of x1,000 and x10,000.

### Statistical analysis

The mean bond strength and standard deviation (SD) of all eight specimens were calculated for each test group. The reliability of the sample size and the assumption of homoscedasticity were confirmed using power analysis and Levene's test, respectively. The data were analyzed using analysis of variance (ANOVA) and a *post hoc* (Tukey-Kramer HSD) test, statistical significance was set at 0.05.

## Results

### Shear bond strength

The results of a two-way ANOVA indicate that the bond strength was significantly influenced by both the primer (P=0.0005) and the adhesive agent (P<0.0001) independently and that their interaction did not reach a level of statistical significance (Table 1).

**Table 1:** Two-way ANOVA results

Source of variation	d.f.	Sum of squares	Mean square	F-value	P-value
Silane primer	3	980.281	326.76	6.266	0.0005
Adhesive agent	5	9,858.206	1,971.641	37.812	<0.0001
Silane primer/ Adhesive agent	15	928.831	61.922	1.187	0.2857
Residual	168	8,759.852	52.142		

**Figure 3:**
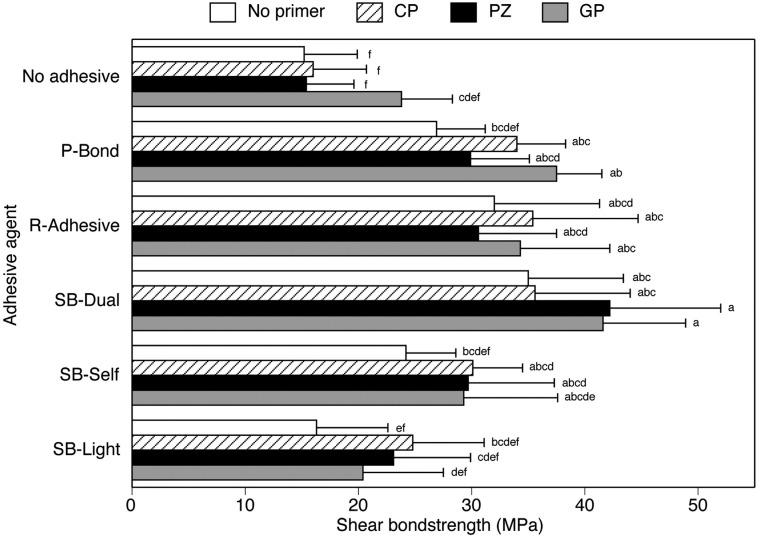
Results of shear bond testing. Identical letters (a, b, c, d, e, f) indicate that the difference between values is not statistically significant (p≥0.05)

The mean bond strength ranged from 15.2 to 42.2 MPa (Figure 3, Table 2). PZ/SB-Dual and GP/SB-Dual presented the greatest bond strength, however no significant differences were observed among 14 groups (PZ/SB-Dual, GP/SB-Dual, GP/P-Bond, CP/SB-Dual, CP/R-Adhesive, No primer/SB-Dual, GP/R-Adhesive, CP/P-Bond, No primer/R-Adhesive, PZ/R-Adhesive, CP/SB-Self, PZ/P-Bond, PZ/SB-Self, and GP/SB-Self). No primer/P-Bond, No primer/SB-Self, and all the SB-Light and No adhesive groups presented the lowest bond strength. Within each adhesive agent group, no statistically significant difference was observed among the No primer, CP, PZ, and GP specimens.

**Table 2:** Shear bond strengths and failure modes observed after shear bond testing

		Mean (SD)* (MPa)			Failure mode** (number of specimens)			
	No primer	CP	PZ	GP	No primer	CP	PZ	GP
No adhesive	15.2 (4.7)^f^	16.0 (6.2)^f^	15.4 (4.2)^f^	23.8 (4.5)^cdef^	Ad(8)	Ad(8)	Ad(8)	Ad(8)
P-Bond	26.9 (4.3)^bcdef^	34.0 (6.0)^abc^	29.9 (5.2)^abcd^	37.5 (4.0)^ab^	Ad(8)	Ad(8)	Ad(8)	Ad(7) Ad/Cr(1)
R-Adhesive	32.0 (9.3)^abcd^	35.4 (11.8)^abc^	30.6 (6.9)^abcd^	34.3 (7.9)^abc^	Ad(4) Ad/Cr(4)	Ad(1) Ad/Cr(7)	Ad(5) Ad/Cr(3)	Ad(4) Ad/Cr(4)
SB-Dual	35.0 (8.4)^abc^	35.6 (9.9)^abc^	42.2 (9.8)^a^	41.6 (7.3)^a^	Ad(5) Ad/Cr(3)	Ad(4) Ad/Cr(4)	Ad(1) Ad/Cr(7)	Ad/Cr(8)
SB-Self	24.2 (4.4)^bcdef^	30.1 (6.3)^abcd^	29.7 (7.6)^abcd^	29.3 (8.3)^abcde^	Ad(8)	Ad(8)	Ad(7) Ad/Cr(1)	Ad(8)
SB-Light	16.3 (6.3)^ef^	24.8 (8.9)^bcdef^	23.1 (6.8)^cdef^	20.4 (7.1)^def^	Ad(8)	Ad(8)	Ad(8)	Ad(8)

* Identical small letters indicate values that are not statistically different (p≥0.05)

** Ad: Adhesive failure at the interface between the composite block and the veneered resin composite, Ad/Cr: Combinations of adhesive failure and crack propagation inside the composite block.

Regarding the mode of failure when using R-Adhesive or SB-Dual, a greater number of specimen failures were in the category of Ad/Cr. Except for two specimens, all the specimens in the No adhesive, P-Bond, SB-Self, and SB-Light specimen groups presented only Ad.

### Scanning electron microscopy

**Figure 4:**
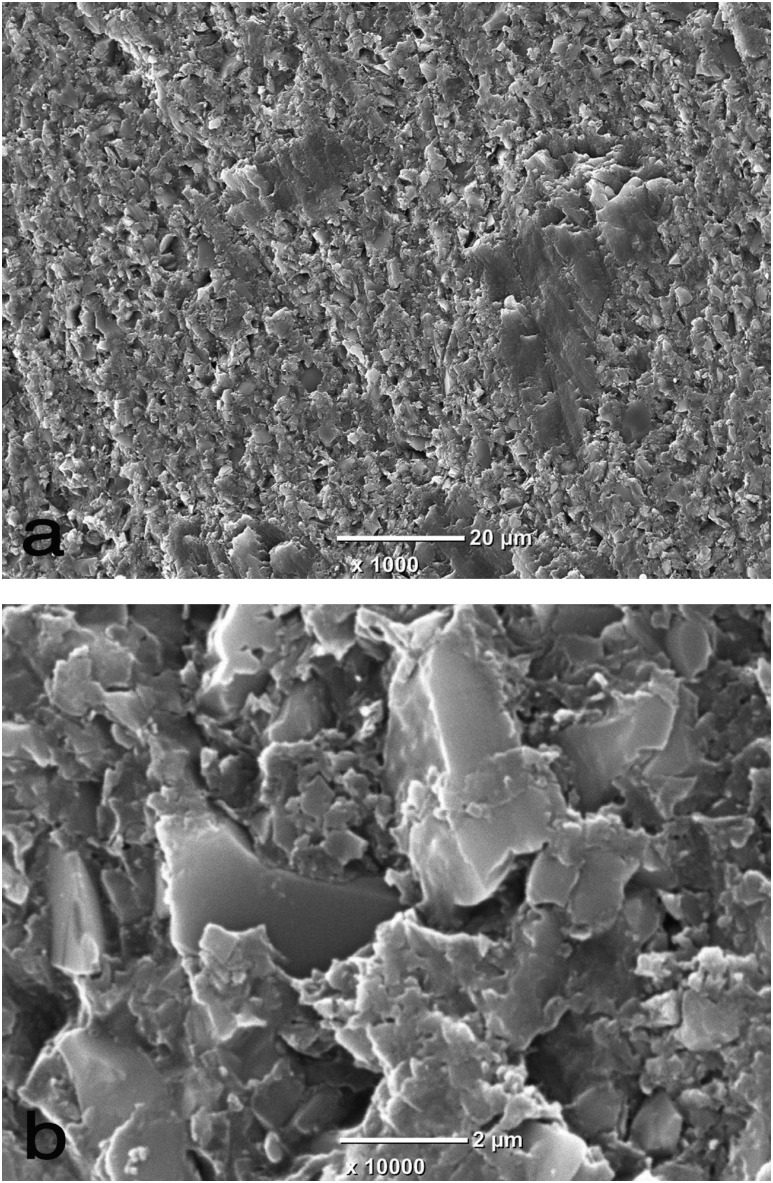
Scanning electron micrograph of a CAD/CAM composite specimen ground with 600-grit silicon-carbide abrasive paper. Original magnification: (a) ×1,000 and (b) ×10,000

Figure 4 shows SEM images of the ground CAD/ CAM composite surface. Numerous supramicron and submicron particles of the hybrid filler can be observed.

The surfaces of the filler particles were relatively smooth against the roughened matrix resin.

## Discussion

This study assessed the effects of three silane primers and five types of coupling agents on bonding between a composite block and a light-curing resin composite. The results of two-way ANOVA indicated that the addition of coupling agents and silane primers made a statistically significant difference on the bond strength. Therefore, the null hypothesis was rejected. ANOVA also indicated that the contribution from coupling agents to the bond strength was greater than the silane primers.

Both GP/P-Bond and GP/R-Adhesive were applied according to the manufacturer's protocol for repairing resin composite restorations. The reported shear bond strength^19^ ^,^ ^22^ between a resin composite and six different CAD/CAM polymer materials were lower than those of GP/P-Bond and GP/R-Adhesive. Therefore, the relatively high bond strength presented by GP/ SB-Dual in this study, is notable when compared to both GP/P-Bond and GP/R-Adhesive. Comparing the molecular weight of methyl methacrylate (MMA) to the molecular weights of the dimethacrylates contained in P-Bond and R-Adhesive, MMA presents low weight. This difference in monomer composition could be one of the factors affecting the diffusion of the coupling agents into the slightly roughened matrix resin (Figure 4).

The different bond strengths generated by the three MMA-based adhesive agents (SB-Dual, SB-Self, and SB-Light) suggest the presence of other factors not related to MMA. SB-Dual contains both a TBB derivative and a photoinitiator. SB-Light (containing no TBB derivative) and SB-Self (containing no photoinitiator) were used as controls to evaluate the role of the TBB derivative. The greater bond strengths obtained from SB-Dual suggest cooperative effects between TBB and the photoinitiator, SB-Self and SB-Light presented lower values. This supports the findings of a previous study, which reports that the combined used camphorquinone and TBB was effective to decrease residual monomer and to promote the postpolymerization of the resin^9^. The degree of conversion of the polymerized Gradia composite, measured by Fourier transform near infrared spectroscopy, is reported to be around 40-50%^7^. Considering this, we speculate that the unpolymerized methacryloyl groups remaining in the Gradia Block get chemically bonded to the methacrylates in the primers and coupling agents by radical polymerization.

The composite block used contained 73% inorganic filler and 3% organic-inorganic composite filler. Silane coupling agents react with inorganic components such as silica to form siloxane bonds; they also copolymerize with methacrylates^2^ ^,^ ^18^. However, in this study we did not obtain a high bond strength using a silane primer alone (CP/No adhesive, PZ/No adhesive, or GP/No adhesive). We hypothesize that the inorganic filler particles had already been treated with silane coupling agents during the manufacture process, therefore, the substrate surface contained few exposed inorganic components. This could be the reason for the limited effect of the silane primers on bond strength.

The mode of failure tended to shift from Ad to Ad/Cr as the bond strength increased (Table 2). R-Adhesive and SB-Dual presented more Ad/Cr, indicating a higher bond strength, than the No adhesive, P-Bond, SB-Self, and SB-Light specimens. Neither the composite block nor the veneering resin composite presented complete cohesive failure, suggesting that the adhesive force was inferior to the cohesive strength of the resin composite materials. Thus, the improvement on the adhesive force seems to be a worthy subject for further research.

Clinically, a number of factors, such as aging of restorative material in the presence of saliva, plaque, pellicle, and biting force, would influence the bond strength. The bond strength testing performed in this study does not reflect all possible contributory factors that might occur in clinical settings. We used a micropipette to control the amount of primer and coupling agent applied on the specimen, clinicians usually use other applicators, such as a mini-sponge or a brush; these different application methods may affect adhesive bonding. However, the 24 h bond strength obtained is useful as a preclinical screening tool. Although long-term clinical observation is needed to validate the findings of this study, practitioners, when repairing or veneering CAD/CAM composite restorations, should consider that strong bonding of a composite block might not be achieved without the use of a coupling agent containing a suitable polymerization initiator.

## Conclusions

Within the limitations of this study, we conclude that the combination of a silane primer (GP or PZ) and a dual-curing adhesive agent (SB-Dual) improves the bond strength between a composite block and a lightcuring resin composite. Furthermore, the contribution of the coupling agent to the bond strength is greater than that of the silane primers.
